# THE INFLUENCE OF LIFE-EXTENDING MUTATION AND DIETARY INTERVENTION ON GUT MICROBIOTA

**DOI:** 10.1093/geroni/igz038.3072

**Published:** 2019-11-08

**Authors:** Michal Masternak, Denise S Wiesenborn, Augusto Schneider, Till Strowig, Karl –Herbert Schafer, Eric Galvez

**Affiliations:** 1 University of Central Florida, Orlando, Florida, United States; 2 Burnett School of Biomedical Sciences, Orlando, Florida, United States; 3 Helmholtz Centre for Infection Research, Braunschweig, Sachsen-Anhalt, Germany; 4 University of Applied Sciences, Zweibrucken, Rheinland-Pfalz, Germany

## Abstract

The gastrointestinal microbiota represents a large and complex ecological system of different microorganisms. Recently, there is an increasing interest in the impact of microbiota on development of different age-related diseases. We tested the changes of gut microbiota during development in long-living Ames dwarf (df/df) mice and we compared the effects of this life-extending mutation with the impact of calorie restriction (CR). Importantly, the analysis of microbiome showed significant differences in the ratio of Bacteroidetes and Firmicutes when comparing df/df and normal (N) mice (p<0.001). The LefSe analysis showed distinct microbiome distribution between CR and ad libitum (AL) feeding regimen in N animals (p<0.004), yet there was lack of similar changes in response to CR in df/df mice. In summary, our study showed significant genotype impact on gut microbiota and we showed that life-extending CR regimen provide divergent effects on gut microbiota in N when comparing with df/df mice.

